# Effectiveness of Interventions to Reduce Tobacco Smoke Pollution in Homes: A Systematic Review and Meta-Analysis

**DOI:** 10.3390/ijerph121215038

**Published:** 2015-12-18

**Authors:** Laura J. Rosen, Vicki Myers, Jonathan P. Winickoff, Jeff Kott

**Affiliations:** 1 School of Public Health, Sackler Faculty of Medicine, Tel Aviv University, P.O.B. 39040, Ramat Aviv 69978, Israel; vicki_myers@hotmail.com; 2 Massachusetts General Hospital and Harvard Medical School, Boston, MA 02451-1137, USA; jwinickoff@mgh.harvard.edu; 3 Sackler School of Medicine, Sackler Faculty of Medicine, Tel Aviv University, P.O.B 39040, Ramat Aviv 69978, Israel; jeffreykott@gmail.com

**Keywords:** tobacco smoke exposure (TSE), environmental tobacco smoke (ETS), home air quality, air nicotine, respirable small particles (RSPs)

## Abstract

*Introduction*: Smoke-free homes can help protect children from tobacco smoke exposure (TSE). The objective of this study was to conduct a meta-analysis to quantify effects of interventions on changes in tobacco smoke pollution in the home, as measured by air nicotine and particulate matter (PM). *Methods*: We searched MEDLINE, PubMed, Web of Science, PsycINFO, and Embase. We included controlled trials of interventions which aimed to help parents protect children from tobacco smoke exposure. Two reviewers identified relevant studies, and three reviewers extracted data. *Results*: Seven studies were identified. Interventions improved tobacco smoke air pollution in homes as assessed by nicotine or PM. (6 studies, *N* = 681, *p* = 0.02). Analyses of air nicotine and PM separately also showed some benefit (Air nicotine: 4 studies, *N* = 421, *p* = 0.08; PM: 3 studies, *N* = 340, *p* = 0.02). Despite improvements, tobacco smoke pollution was present in homes in all studies at follow-up. *Conclusions*: Interventions designed to protect children from tobacco smoke are effective in reducing tobacco smoke pollution (as assessed by air nicotine or PM) in homes, but contamination remains. The persistence of significant pollution levels in homes after individual level intervention may signal the need for other population and regulatory measures to help reduce and eliminate childhood tobacco smoke exposure.

## 1. Introduction

Protection of nonsmokers from tobacco smoke exposure (TSE) in indoor public places is regulated by law in many countries [1]. However, the home environment, where children and adults spend much of their time, is a private sphere which is generally unregulated. Some research suggests that while bans on smoking in public places have greatly reduced overall exposure in public places in many countries, the home is now becoming the primary source of tobacco smoke exposure [2], from both secondhand and thirdhand smoke [3]; this has occurred despite the fact that bans on smoking in public places have not displaced smoking to the home, and are in fact associated with decreases in child exposure to tobacco smoke [4,5,6].

Roughly 40% of children worldwide are exposed to the harmful effects of TSE [7]; many of these children are “captive” smokers in their own homes. Exposure of children increases the risk for many health problems including lower respiratory infection, asthma, and acute otitis media [7], sudden infant death syndrome, compromised lung function, school absenteeism and days of restricted activity [1,7,8,9,10]. Detrimental health effects of TSE persist into adulthood [8,11], with children of smoking parents at greater risk for tobacco use [12].

One obvious route to attaining smoke-free homes is for all family members to be nonsmokers and to have a strict ban on smoking in all places in the home for all residents and visitors. However, family members who are smokers may be heavily addicted [13], and intervention trials aimed at convincing parents to quit smoking for the benefit of their children fail with the great majority of intervention trial participants [14,15]. Smoke-free homes provide a harm-reducing, voluntary solution: while they are unlikely to entirely protect children of continuing smokers, they have to potential to greatly reduce the exposure of children and its attendant harm, without requiring that parents quit smoking.

Indeed, some investigators have developed programs which focused on reducing child exposure to tobacco smoke, rather than quitting; a meta-analysis of these trials, which examined outcomes of behavioral change by parents or biochemical measures of exposure in children, showed some evidence of benefit [16].

However, measurement of effectiveness of interventions to protect children from tobacco smoke exposure is challenging. Parental reports of child exposure may be inaccurate [17], and some parents may be averse to collection of biomarkers from their children [18]. Home air quality, on the other hand, holds the potential to provide an accurate and non-invasive proxy measure of exposure. Air nicotine and particulate matter (PM) are recognized measures of secondhand tobacco smoke which have been widely used to measure indoor tobacco smoke air pollution in homes and workplaces [19]. While air nicotine is specific to tobacco smoke, PM can be elevated by multiple sources; however, studies have shown dramatic increases in PM caused by cigarette smoking in the home [2]. The aim of this study was to conduct a systematic review and meta-analysis of intervention trials which aimed to reduce child TSE, in order to determine whether these interventions had any effect on home tobacco smoke air pollution as measured by air nicotine or PM.

## 2. Experimental Section

### 2.1. Data Sources and Strategy

PRISMA reporting guidelines for meta-analysis were followed [20] (see the Supplementary Material Table S1 for a checklist). We conducted a targeted search of the literature with the aid of a librarian specializing in medical databases (Ruth Suhami). We searched Medline (Ovid), PubMed, PsychINFO, Web of Science, and Embase in October 2013 for all relevant studies, regardless of publication date, and performed an update in July 2014. We created the search strategy in Medline and then adapted it to all other databases, changing vocabulary and syntax as appropriate for each database. We used both index terms and keyword searches as follows:

(1) Medline: We used the following MESH terms: Tobacco Smoke Pollution OR (Parents AND “Tobacco Use Cessation”).
(2) Embase: We used the following EMTREE terms: second hand smoke OR passive smoking OR (smoking cessation AND parents).
(3) PsycINFO: We used the Index Term “Passive Smoking”.
(4) All databases: We searched for the following keywords: second-hand smoke OR passive smoking OR environmental tobacco smoke OR involuntary smoking OR Tobacco smoke exposure (with all variations of spellings and endings).

Inclusion was limited to papers published in English. Because we were primarily interested in tobacco smoke air pollution as measured by air nicotine or PM in homes with young children, we limited all searches to the age groups of newborn/infant/children (ages 0–12).

In order to obtain high-quality studies with a comparable control group, we used a randomized controlled trial (RCT) filter for the search, and every article had to be a randomized controlled trial or a controlled clinical trial. We excluded the following publication types: case-control study, cross-sectional study, meta-analysis, systematic review, protocol, observational study, and guideline. We required follow-up of at least one month from the beginning of the trial in order to avoid focusing on immediate outcomes, which we hypothesized might be temporary in nature. In cases in which data on relevant outcomes were collected but required information was missing, we attempted to contact study authors for data.

### 2.2. Data Extraction

Relevant studies were independently identified by two authors (L.J.R. and J.R.K.). Data were independently extracted by three authors (L.J.R., J.R.K. and V.M.) and then compared. Differences were resolved through discussion.

### 2.3. Methodological Quality

We assessed study design, blinding of observers, percent follow up, fidelity to treatment, and whether the control group received an active intervention.

### 2.4. Study Eligibility

To be included, the studies had to meet the following criteria:

(1) Study design: RCT using a cluster or individual randomization scheme, quasi-randomized RCT, or controlled trial (CT).
(2) Participants: Parents (mother, father, or both parents) or caregivers of children between 0 and 12 years.
(3) Types of interventions: Any type of intervention that had as one of its aims helping parents decrease TSE of their children.
(4) Length of observation period: Minimum 1 month from start of intervention. In studies reporting different follow-up times, we used the longest available period.
Relevant outcome: Measurement of air nicotine or particulate matter.
(6) Availability, in paper or from the author, of means, standard deviations, and n’s for change in tobacco smoke air pollution or tobacco smoke air pollution at study end.

### 2.5. Outcomes

Our primary interest was in change in tobacco smoke air pollution from baseline to longest follow-up, as measured by air nicotine or particulate matter (PM). Tobacco smoke air pollution at study end was a secondary endpoint. We included studies which reported on home air nicotine in any form (raw values, log values, or geometric means), or particulate matter (PM) in any form (raw values, log values, or geometric means).

### 2.6. Data Analysis

We used meta-analysis to compare intervention and control groups regarding (1) changes in home tobacco smoke air pollution, from pre-intervention to longest follow-up; and (2) home tobacco smoke air pollution at end of the trial (longest follow-up). We conducted three meta-analyses for each outcome, as follows: (a) an analysis which included air nicotine levels, if available, and PM if air nicotine was not available; (b) an analysis which included air nicotine only; and (c) an analysis which included PM only.

### 2.7. Other Variables

We extracted data on the following additional variables:

Study-related: Child cohort, recruitment setting, provider, number of sessions in intervention, observation times, measures of air quality, exposure assessment duration, Intervention components: Self-help materials, counseling, phone support, nicotine replacement therapy (NRT), biochemical feedback, air cleaner, feedback on air quality.

### 2.8. Meta-Analytic Approach

Statistical analyses and meta-analyses were performed using RevMan 5.2.7.(Cochrane Collaboration: Copenhagen, Demark) We used the DerSimonian and Laird random-effects method with 95% CIs to pool results. [21] We chose to use the random-effects method because we assumed that different intervention conditions would be associated with different effects, and we were interested in getting an average of the true effects from the population of intervention studies (as opposed to an estimate of a single population effect, as would be the case were we to use the fixed-effects method) [22].

Because the outcomes measured differed both in scale and in presentation (raw values, log values, geometric means), we used the standardized mean difference (SMD) rather than mean difference for estimation of intervention effectiveness. Estimates are presented with 2-sided 95% CIs. The pooled SMDs were estimated with weights based on the inverse variance method and adjusted for the random effects assumption [22].

## 3. Results

### 3.1. Description of Studies

**Figure 1:**
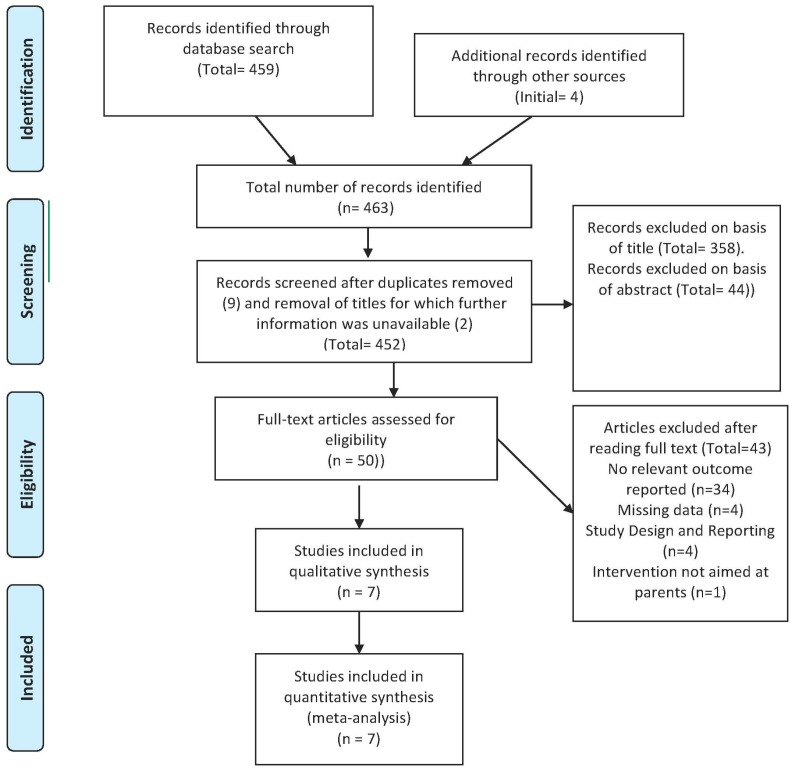
Flowchart for identification of studies.

Our initial targeted, systematic search, conducted in October of 2013 and updated in July of 2014, identified 459 records. We were aware of four additional studies from previous work in the field. Nine records were duplicates, and we were unable to find information on two identified records. We scanned the titles of 452 articles. Most articles (*n* = 358) were excluded on the basis of title, while another 44 articles were excluded after reading the abstract. Overall, 50 articles were read in full. *Of* these 43 were excluded for the following reasons: no objective measure of home air quality, 34 studies [23,24,25,26,27,28,29,30,31,32,33,34,35,36,37,38,39,40,41,42,43,44,45,46,47,48,49,50,51,52,53,54,55,56]; missing data (means and/or standard deviations and/or samples sizes), four studies [57,58,59,60]; study design and reporting: four studies (not reporting on RCT/CT results: one study [61], not a report of an RCT: one study [62]; no true control group: two studies [63,64] not aimed at parents for child TSE protection, one study [65]. We used published and unpublished data supplied by authors for seven relevant studies [66,67,68,69,70,71,72]. The seven included trials were published between 2009 and 2014. All except one [69], which was run in Scotland, were done in the continental United States. A flowchart describing the identification process is presented in Figure 1. Study characteristics of included trials are presented in Table 1 and Table 2.

**Table 1:** Characteristics of included studies.

Study	Location	Child Cohort	Recruitment Setting	Provider	Number of Sessions	Observation of Outcome of Interest	Measures of Air Quality(Instrument)	Exposure Assessment Duration	Intervention Components Other
Butz 2011 [72]	Baltimore	Asthmatic	Hospital/Physician rosters of asthmatic children	Nurse health coaches	4 home visits	0, 6 months	Air Nicotine (Passive dosimeter) PM_2.5_ and PM_10_ (MSP impactor *)	7 days	B, F
Eakin 2014 [70]	Baltimore	Well	Head Start (development program for low-income families)	Health counselors	5 home visits	0, 3, 6, 12 months	Air Nicotine (passive dosimeter)	7 days	A, B, E
Hovell 2009 [71]	San Diego	Well	Supplemental Nutrition Program for Women, Infants and Children (low-income families)	Study Counselor	10 home visits, 4 phone calls	0, 3, 6, 12, 18 months	Air Nicotine (Nicotine dosimeter **)	7 days	A, B, C, D
Lanphear 2011 [66]	Cincinnati	Asthmatic	Hospital, clinic, or emergency room	Project staff	1 home visit to install air cleaners	Nicotine: 6, 12 months PM: 0, 6, 12 months	Air Nicotine (passive dosimeter) PM > 0.3 µm PM > 5µm (GT-321 particle counter, Met One ***)	Air nicotine: 6 months, PM: 1 minute	F
Prokhorov 2013 [67]	Houston	Well	Cohort of Mexican households from existing database	Project staff	Baseline home visit for provision of materials	0, 6, 12 months	Air Nicotine (Passive dosimeter)	7 days	A
Stotts 2013 [68]	Houston	Babies in NICU with high respiratory risk	Hospital-NICU	MI Counselor	2 face-to face sessions at hospital	1, 6 months	Air Nicotine (Passive dosimeter)	2 weeks	A, B
Wilson 2013 [69]	Aberdeen	Well	General practitioner practices	Researcher	4 home visits	0, 4 weeks	PM_2.5_ (Sidepak monitor †)	24 h	B, E, G

A: self-help materials; B: counseling; C: phone support; D: nicotine replacement therapy (NRT); E: biochemical feedback; F: Air cleaner; G: Tobacco smoke air pollution feedback (Air nicotine or PM); PM, particulate matter; MI, motivational interviewing; NICU, neonatal intensive care unit. *, MSP, St Paul, MN, USA; ** Pall, Putnam, CT; *** Met One: Oregon, OR, USA; †, TSI, MN, USA

**Table 2:** Methodological characteristics of included studies.

Study	*N*	Design	Blinding of Observers	Percent Follow-Up *N* (%) (Change Analysis)	*N* (%) Participants Received Full Intervention in Treatment Group (Fidelity)	Control Group Intervention (During Or After Study)
Butz, 2011 [72]	85 ^a^	RCT	Yes	80 (94%)	NR	Asthma Education
Eakin, 2013 [70]	330	RCT	NR	235 (71%)	54 (33% attended 4 sessions)	Education
Hovell, 2009 [71]	50 ^b^	RCT	Yes	32 (64%)	41 (54%)	Usual Care + Self-help materials at end of study
Lanphear, 2011 [66]	225	RCT	Yes	214 (95)%	110 (100%)	Inactive Air Cleaners
Prokhorov, 2013 [67]	91	RCT	NR	74 (81%)	NR	Self-help for quitting
Stotts, 2013 [68]	110 ^c^	RCT	NR	NA	44 (71%)	Usual hospital care, including advice at discharge about SHSe dangers and advice to not smoke around infant or quit smoking
Wilson, 2013 [69]	59	RCT	NR	46 (78%)	21 (70%)	Counseling

^a^. Total in trial was *N* = 126. We used two of three groups: air monitoring + coach, and control; ^b^. Total in trial was *N* = 150. One third of these (*N* = 50) were chosen to receive active nicotine air dosimeters. ^c^. Total in trial was *N* = 144. We used two of three groups: intervention + reduced measurement usual care control. NR: Not relevant.

### 3.2. Intervention Arms and Components

Five studies had one intervention group and one control group [66,67,69,70,71]; while two studies had multiple intervention arms [68,72]. Of the two intervention arms in Butz *et al.* [72] one group was provided only air cleaners and one had air cleaners plus a health coach. We used the air cleaner and health coach group as our specified intervention arm and the control group as control. Stotts *et al.* [68] had two control groups: usual care and usual care-reduced measurement. We used the reduced measurement group as our specified control. One study reported air nicotine measurements in multiple settings—A higher exposure setting and a lower exposure setting [67]. In this instance, the data provided for the higher exposure setting was used.

Interventions included the following components: counseling, five studies [68,69,70,71,72]; phone support, one study [71]; nicotine replacement therapy (NRT), one study [71]; biochemical feedback, two studies [69,70]; air cleaners, two studies [66,72]; self-help materials, four studies [67,68,70,71]; tobacco smoke air pollution feedback (air nicotine or PM), one study [69].

### 3.3. Intervention Intensity

Intervention intensity ranged from one visit to install air cleaners [66] or provide self-help materials [67] to 10 home visits and four phone calls [71]. Three of the studies included 4–5 home visits [69,70,71], and one study included two face-to-face in-hospital sessions [68].

### 3.4. Blinding

Three studies reported blinding of observers [63,66,68,69,71,72]. The remaining studies did not report on blinding of observers.

### 3.5. Fidelity

Four studies reported on fidelity. Of these, one reported 100% fidelity to treatment [66], two studies reported approximately 70% of participants having received the entire intervention [68,69], while the fourth study reported lower fidelity of 54% [71]. The remaining studies did not report how many participants received all intervention elements, although one study reported that 33% attended four out of five sessions [69].

### 3.6. Control Group Intervention

In five of the studies, the control group participants received some kind of intervention pertaining to smoking, cessation, or risk to children from smoking [67,68,69,70,72]. In one study, control group participants received related self-help materials at the close of the study [71] while participants in one study received inactive air cleaners [66].

### 3.7. Outcome Measures

Of the three studies examining PM, two measured PM_2.5_ (particles < 2.5 µm) [69,72], while one study measured particles between 0.3 and 5 µm [66].

Air nicotine was measured for 1 week at each time point in most studies [67,70,71,72], for 2 weeks in one study [68], and for 6 months in one study [66]. PM measurements were averaged over 7 days [72] 1 min [66] or 24 h [69]. 

Where specified, analysis was done by gas chromatography. Limits of detection varied: Butz [72] used 0.003 ng/m^3^, Stotts [68] used 0.02 ng/m^3^, and Eakin [70] and Lanphear [66] used 0.01 ng/m^3^ Eakin [70] collected data in two locations and used the mean of the two monitors in her analysis. She also collected a blank and a duplicate for every 10 samples, using a random sampling procedure.

### 3.8. Intervention Effects

**Figure 2:**
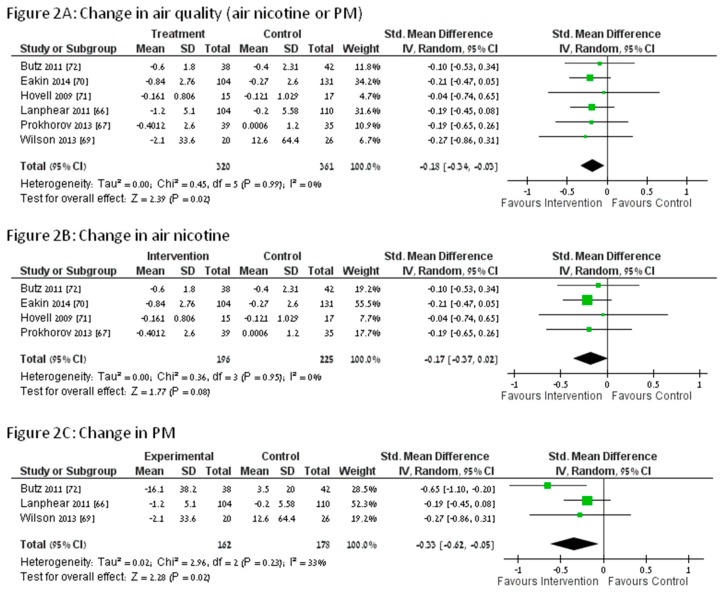
Change in (**A**) air quality; (**B**) air nicotine and (**C**) PM.

**Figure 3:**
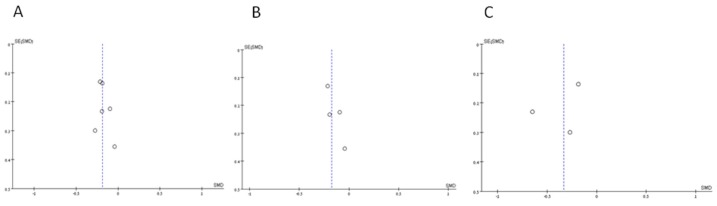
Plots to assess publication bias. (**A**) Combined air nicotine or PM; (**B**) Air nicotine; (**C**) PM.

The forest plots for the meta-analyses of the primary outcome, change in air quality, can be seen in Figure 2. The respective publication bias plots for each analysis are shown in Figure 3. Table 3 presents changes in PM and air nicotine from baseline to follow up. Of the seven studies, six reported air nicotine levels [66,67,68,70,71,72] while three reported PM levels [66,69,72]. Tobacco smoke air pollution improved in all intervention groups and some control groups for which baseline data were available as measured by nicotine and by PM (Table 1).

**Table 3:** Tobacco smoke air pollution (air nicotine and/or PM) in intervention and control groups, by time: Particulate matter and Air nicotine in intervention and control groups.

			Intervention			Control		
Study	Longest Endpoint	Outcome	Baseline Mean ± STD; *N*	Follow-Up Mean ± STD; *N*	Change Mean ± STD; *N*	Baseline Mean ± STD; *N*	Follow-Up Mean ± STD; *N*	Change Mean ± STD; *N*
Butz 2011 [72]	6 months	PM_2.5_	45.4 ± 34.7; *N* = 41	32.2 ± 30.1; *N* = 38	−16.1 ± 38.2; *N* = 38	39.5 ± 24.1; *N* = 44	38.9 ± 25.0; *N* = 42	3.5 ± 20.0; *N* = 42
		Air nicotine	1.4 ± 1.7; *N* = 41	0.9 ± 1.1; *N* = 38	−0.6 ± 1.8; *N* = 38	1.8 ± 2.8; *N* = 44	1.4 ± 2.04; *N* = 42	−0.4 ± 2.31; *N* = 42
Eakin 2014 [70]	12 months	Air nicotine	2.10 ± 3.05; *N* = 164	1.15 ± 1.77; *N* = 105	−0.84 ± 2.76; *N* = 104	1.52 ± 2.25; *N* = 164	1.29 ± 2.45; *N* = 132	−0.27 ± 2.60; *N* = 131
Hovell 2009 [71]	6 months	Air nicotine	0.907 ± 0.709; *N* = 26	0.853 ± 0.942; *N* = 17	−0.161 ± 0.806; *N* = 15	1.099 ± 0.945; *N* = 24	0.708 ± 0.613; *N* = 19	−0.121 ± 1.029; *N* = 17
Lanphear 2011 [66]	12 months	Particles >0.3, geometric means	4 ± 2.56; *N* = 110	3.0 ± 2.61; *N* = 104	−1.2 ± 5.10; *N* = 104	4.7 ± 2.58; *N* = 115	4.4 ± 2.65; *N* = 110	−0.2 ± 5.58; *N* = 110
		Air nicotine, geometric means	Baseline data not collected	2.5 ± 7.65; *N* = 110	NR	Baseline data not collected	2.7 ± 7.78; *N* = 115	NR
Prokhorov 2013 [67]	12 months	Air nicotine—High exposure Room	1.14 ± 2.5; *N* =47	0.20 ± 0.53; *N* = 39	−0.4012 ± 2.6; *N* = 39	0.55 ± 0.84; *N* = 42	0.17 ± 0.29; *N* = 35	0.0006 ± 1.2; *N* = 35
Stotts 2013 [68]	6 months	Air nicotine	Baseline data not collected	0.2088 ± 0.3256; *N* = 34	NR	Baseline data not collected	0.5075 ± 1.181; *N* = 20	NR
Wilson 2013 [69]	1 month	Geometric mean PM_2.5_	19 ± 3; *N* = 25	11 ± 4; *N* = 20	−2.1 ± 33.6; *N* = 20	25 ± 3; *N* = 28	24 ± 5; *N* = 27	12.6 ± 64.4; *N* = 26

NR: Not Relevant.

Change in air quality, our primary endpoint, was greater in the intervention groups than the control groups (six studies, *N* = 681, Standardized Mean Difference (SMD) = −0.18, Confidence Interval (CI): [−0.34, −0.03], *p* = 0.02). Analysis of change in PM alone showed a benefit to the intervention group (three studies, *N* = 340, SMD = −0.33, CI: [−0.62, −0.05], *p* = 0.02), while analysis of change in air nicotine alone showed a trend towards a benefit to the intervention group (air nicotine change: four studies, *N* = 421, *p* = 0.08). The secondary end-of-study analysis showed a non-significant trend towards benefit among intervention participants (seven studies, *N* = 753, SMD = −0.38 CI: [−0.81, 0.05], *p* = 0.08). PM, but not air nicotine, was significantly lower at study end among intervention group participants (PM: three studies, *N* = 341, SMD = −1.10, CI: [−2.12, −0.07], *p* = 0.04; Air nicotine: six studies, *N* = 706, *p* = 0.32).

## 4. Discussion

The present meta-analysis demonstrates that interventions to protect children from tobacco smoke exposure improve home tobacco smoke air pollution (air nicotine and/or PM), but only to a limited degree: some pollution was still present in homes in all studies post-intervention. The included studies showed reductions in air nicotine and PM at follow-up in intervention compared to control groups, but children were still being exposed to tobacco smoke to some degree, with no intervention entirely eliminating exposure or fully protecting children. While PM levels may have been affected by environmental exposures other than smoking, air nicotine is tobacco-specific and therefore any amount above zero is indicative of smoking in the home, or the entry into the home of smoke produced outside of the home, through windows, doors, ventilation systems, or other means.

International air quality standards provide a context for these findings. The US Environmental Protection Agency standard for 24 h PM_2.5_ pollution is a maximum level of 35 µg/m^3^ [73]; the World Health Organization’s threshold is lower, at 25 µg/m^3^ (24 h mean) [74]. In the Butz 2011 [72] study, average baseline values in both intervention and control groups were higher than the WHO and EPA cut-offs (Intervention: 45.4, Control: 39.5). At follow-up, the intervention group fell substantially to 32.2—Below the EPA standards, but above the WHO standards, while the control group averaged 38.9 at study end, higher than the EPA and WHO standards. The intervention which Butz used was a combination of air cleaners and motivational interviewers. The finding of continuing contamination after use of air cleaners is consistent with the finding of the U.S Surgeon General; the 2006 report states that “… exposure to secondhand smoke components cannot be controlled sufficiently through dilution ventilation or by typical air cleaning strategies, if the goal is to achieve no risk or a negligible risk” [9]. It was not possible to compare the results of Lanphear [66] or Wilson [69], the other two studies which reported on PM, with the international standards, because Lanphear collected information on PM_3_, not PM_2.5_, and presented geometric means, while Wilson presented geometric means of PM_2.5_, which are not comparable with the untransformed numbers.

There are no equivalent air quality standards for nicotine, since no level is considered safe. However, some information can be obtained from Wipfli’s [75] study, which assessed home air nicotine in smoking and non-smoking families in 31 countries; she reported a median value of 0.18 µg/m^3^ in households with smokers, compared to 0.01 µg/m^3^ in households without smokers. We had access to raw data on air nicotine for five studies [67,68,70,71,72]. Average baseline values ranged from 0.55 to 2.1, and average values at follow-up ranged from 0.20 to 1.15 in the intervention groups, and 0.17 to 1.4 in the control groups. These numbers suggest very high levels of indoor exposure prior to, and after, completion of the interventions in both intervention and control groups, despite statistically significant reductions over the course of the trials in the intervention versus control groups.

### 4.1. Intervention Components and Effects

The interventions included in the review were implemented using diverse tools, from the behavioural (face-to-face or phone counselling; biochemical feedback from child biomarkers or tobacco smoke air pollution measures (air nicotine or PM); comic books and stories with photographs; smoking cessation aids for parents) to the purely technical (air cleaners). Intensity ranged greatly, from a single visit [66] to 10 visits and 4 phone calls [71].

As can be seen from Figure 2, all studies similarly demonstrated at least a trend to benefit among intervention group participants; differences in results were due to the estimated size of the effects and *p*-values, but not direction. The Butz study [72], which targeted children with asthma and employed air cleaners and health coaching, demonstrated a greater reduction in PM in the intervention versus the control group. Eakin [70] and Prohkorov [67], using multivariable methods, (Eakin: generalized estimating equations; Prohkorov: mixed model regression), reported statistically significant reductions in air nicotine in intervention groups compared to control groups. Eakin’s intervention was an intensive intervention, requiring five visits, while Prokhorov’s intervention required only one visit, and was primarily a self-help intervention. The small number of studies and diversity of intervention approaches precluded formal subgroup analyses, and also precluded definitive conclusions about the most effective interventions.

### 4.2. The Importance of, and Challenges to, Measurement and Reporting of Home Air Quality

Given the importance of smoke-free homes in protecting children from tobacco smoke exposure, objective measurement of home tobacco smoke air pollution is necessary. First, direct measurement of home smoke pollution can be used to provide objective feedback to parents, in order to help them internalize the existence of smoke pollution in their homes, and so establish whether it is a safe environment. Second, tobacco smoke air pollution assessment allows an objective measure of intervention effect, particularly if pollution is measured before and after the intervention, and if the study has a randomized control group. Third, objective measures of tobacco smoke air pollution might help to guide the next action steps for an intervention that helps parents to reduce smoking around the home, but does not change child biomarkers of exposure. Knowing more about objective home exposure levels might help quantify modifiable exposures in the home versus exposure elsewhere.

However, the methods used to measure air nicotine and particulate matter across studies in this review, and reporting formats, showed a lack of consensus. The most important issue concerns the decision to measure either air nicotine or PM. Air nicotine has the critical advantage of being a “sensitive and specific indicator for secondhand smoke” [9]. Air nicotine has the disadvantage of requiring laboratory analysis, which is expensive and precludes providing immediate feedback to parents. PM, by contrast, is non-specific to tobacco smoke, and can be affected by many sources such as cooking fumes, traffic pollution, or dust from building work [76]. Wilson [69] considered the provision of immediate PM feedback to be critical to her intervention: the abstract states “the qualitative findings showed that mothers were shocked by the values measured in their homes” Another advantage of the current monitors used to measure PM (Sidepaks and Dylos monitors) is that they provide feedback on a per-unit-time basis (per-second or per-minute), unlike current air nicotine monitors, which provide a simple summary value over a period of time. 

The length of tobacco smoke air pollution assessment differed between studies. Nicotine dosimeters, used to assess air nicotine, were left in homes for the time periods of 7 days [67,70,71,72], two weeks [68], and six months [66]. Whether the sensitivity of dosimeters is retained for long periods of time is unknown; therefore measurement of different time periods may have differential inherent levels of “noise” or accuracy. The increase in non-systematic error tends to bias results towards the null; as such, increased variability could obscure true differences between intervention arms. The difference in length of PM measurements was also marked, with the three studies respectively presenting averages of 7 days’ [72], 24 hours’ [69] or just 1 minute’s exposure [66].

Other differences in measurement were apparent as well. The limit of detection (LOD) of air nicotine differed between studies, as did the size of particulate matter assessed: studies measured PM_2.5_ [67,72], PM_10_ [72], PM > 0.3 μm [66], and PM > 5 μm [66]. We included results for PM_2.5_ where available, or the closest measure.

There were also differences in reporting, with some authors presenting raw data, and others presenting transformed data (most commonly geometric means). Differences in laboratory analysis technique could further thwart comparison between studies, raising questions about how standardized these procedures are.

### 4.3. Challenges in Assessing Intervention Effects

In addition to the challenges of accurate tobacco smoke air pollution measurement, assessment of the effects of interventions is also problematic. As in many behavioural intervention studies, not all participants were fully compliant to study protocol (reported compliance 33%–100%). In this review, this is complicated by the fact that compliance with some types of interventions (turning on an air cleaner) was far simpler than compliance with other types of interventions, which involved difficult behavioural changes by the parents. It is possible that the interventions would have shown greater benefit had fidelity to protocol been higher.

Similar to effects seen in other reviews of behavioural interventions [12,14], parallel reductions in tobacco pollutants in this review were seen in all intervention and many control groups. This simultaneous improvement in control groups is not surprising given that in most studies (all but Lanphear [66] and Hovell [71]), the control group received some form of smoking-related materials or low-intensity intervention during the study period. Indeed, even without related intervention elements, trial participation alone may affect outcome: Hovell *et al.,* reported that up to two thirds of the effect detected in one of his trials may have been due to measurement alone [71]. Baxi also noted improvements in intervention and control groups in her review [12], and suggested the change may have been due to decreasing smoking rates in Western countries. These concurrent changes in intervention and control arms of trials make detection of the true effects of the intervention more difficult, and also underline the importance of measuring levels of tobacco smoke air pollution prior to the provision of the intervention, as well as at the end of the study. 

### 4.4. Strengths and Limitations of the Review

The strengths of this study include: a librarian-aided systematic search of the literature, quantitative synthesis of the individual study results; use of standardized mean differences to control for different sources and scales of air quality; and use of a random effects meta-analytic approach, which produces an estimate of the average of the true effects from the population of intervention studies, and does not assume a single underlying true effect. All included studies were randomized controlled trials, and the six trials in the main change analysis had complete data on between 64% and 95% of participants. Our primary analysis, which examined change from baseline in the treatment and control groups, was not dependent on the standard assumption of equality of groups prior to the intervention. This was particularly important in light of the fact that the number of included studies was modest. A contributor to the relatively small number of identified studies was that some authors reported on medians and interquartile ranges, but not the means and standard deviations (of end of study values, and for change in values during study) which were necessary for inclusion in the meta-analysis; this led to exclusion of four studies from this review, subsequent to unsuccessful attempts to contact the authors for additional information [57,58,59,60]. However, these studies also showed reductions in air nicotine from baseline, indicating that the principal result and direction of this meta-analysis would not have changed had these studies been included. We were unable to assess the differential effects of varying interventional approaches due to the limited sample size. Finally, the main outcome in many studies was PM, which is not specific to tobacco smoke.

### 4.5. Recommendations

Differences in approaches and scales for measures of tobacco smoke air pollution among studies in this review, which included air nicotine and/or PM, suggest that improvement and standardization of measures for evaluation and reporting of home tobacco smoke air pollution—by air nicotine, PM, or other means is needed. Several approaches are possible. A real-time monitor of air nicotine, similar to the Sidepak and the Dylos machines which measure PM, would combine the benefits of specificity of the passive nicotine dosimeter with the benefits of the Sidepak and Dylos machines. These benefits include immediate feedback, without the lag-time of weeks or months until laboratory analyses are available; provision of results on a continuous (per-unit time), not just cumulative basis; and probably lower cost (at least for the Dylos, which is far less expensive than the Sidepak), as there is no need for laboratory analysis and transport charges. Immediate feedback was believed to be very important by authors of a recent study, who described “shock” among mothers who saw the minute-by-minute graphs of PM levels in their homes [69]. Development of such a nicotine-sensitive monitor is currently underway [77], but is not yet commercially available. Another promising approach uses audio-visual feedback on PM [64,78]. Using audio-visual feedback combined with tobacco-specific real-time nicotine monitors currently under development could form the basis for immediate, low-cost, and tobacco-smoke specific feedback. In all cases of feedback, naturally, it is important to provide context and interpretation to the families.

Beyond interventions in families, regulatory approaches such as smoke-free multi-unit housing [79,80] are currently being implemented in some areas to help protect children living in units of shared apartment buildings, both children of smokers in their own homes, and children of non-smokers exposed to neighbors’ cigarette smoke. A further possible public health strategy is the use of media education campaigns, as recommended by the U.S. Institute of Medicine for ending the tobacco epidemic [81]. Use of new media may present an important and low-cost platform for dissemination of information on the benefits of smoke-free homes and cars.

Researchers could facilitate scientific progress on smoke-free homes by ensuring that outcomes be measured prior to commencement of the intervention, at the end of the intervention, and at longer-term follow-up. Reporting of mean changes from baseline to study end, and at longer-term follow-up, and standard deviations of change, for each intervention arm would facilitate future meta-analyses based on change from baseline. Means and standard deviations of endpoints, not just medians and ranges, should be reported for all time points, facilitating comparisons with international standards and inclusion in future meta-analyses.

In summary, a combination of effective actions are necessary to protect children from the damaging and sometimes deadly effect of tobacco smoke in their own homes. First, all parents should be encouraged to quit smoking for the benefit of their children. Second, it is crucial to further develop and disseminate effective interventions to promote smoke-free homes. Third, effects of regulatory approaches such as smoke-free multi-unit housing which have been proposed and are currently being implemented to help protect children living in shared apartment buildings could be implemented widely [81]. Fourth, public health education campaigns could help encourage single-unit family smoke-free housing in cases that may be beyond the reach of regulatory approaches. More generally, decreasing tobacco dependence of society and individuals through compliance with MPOWER recommendations, denormalization, and innovative approaches would surely benefit the health of children.

## 5. Conclusions

The few studies which reported on changes in particulate matter or air nicotine following interventions to protect children from tobacco smoke suggest that such programs are effective in reducing tobacco smoke pollution in homes. However, significant contamination remains, suggesting that other strategies are necessary to fully protect children.

## Supplementary Files

Supplementary File 1
